# Improved Cas9 activity by specific modifications of the tracrRNA

**DOI:** 10.1038/s41598-019-52616-5

**Published:** 2019-11-06

**Authors:** Tristan Scott, Ryan Urak, Citradewi Soemardy, Kevin V. Morris

**Affiliations:** Center for Gene Therapy, City of Hope – Beckman Research Institute and Hematological Malignancy and Stem Cell Transplantation Institute at the City of Hope, 1500 E. Duarte Rd., Duarte, CA 91010 USA

**Keywords:** Genetic engineering, Gene expression, Mutation

## Abstract

CRISPR/Cas is a transformative gene editing tool, that offers a simple and effective way to target a catalytic Cas9, the most widely used is derived from *Streptococcus pyogenes (Sp*Cas9), with a complementary small guide RNA (sgRNA) to inactivate endogenous genes resulting from insertions and deletions (indels). CRISPR/Cas9 has been rapidly applied to basic research as well as expanded for potential clinical applications. Utilization of *sp*Cas9 as an ribonuclearprotein complex (RNP) is considered the most safe and effective method to apply Cas9 technology, and the efficacy of this system is critically dependent on the ability of Cas9 to generate high levels of indels. We find here that novel sequence changes to the tracrRNA significantly improves Cas9 activity when delivered as an RNP. We demonstrate that a dual-guide RNA (dgRNA) with a modified tracrRNA can improve reporter knockdown and indel formation at several targets within the long terminal repeat (LTR) of HIV. Furthermore, the sequence-modified tracrRNAs improved Cas9-mediated reduction of CCR5 surface receptor expression in cell lines, which correlated with higher levels of indel formation. It was demonstrated that a Cas9 RNP with a sequence modified tracrRNA enhanced indel formation at the CCR5 target site in primary CD4+ T-cells. Finally, we show improved activity at two additional targets within the HBB locus and the BCL11A GATA site. Overall, the data presented here suggests that novel facile tracrRNA sequence changes could potentially be integrated with current dgRNA technology, and open up the possibility for the development of sequence modified tracrRNAs to improve Cas9 RNP activity.

## Introduction

CRISPR/Cas9 has emerged a powerful genome editing tool that requires a CRISPR RNA (crRNA) with a interchangeable 20 nt complementary sequence to a target DNA site, and a trans-activating crRNA (tracrRNA) scaffold recognized by a catalytically active Cas9 protein^1,2^. The crRNA can be annealed to the tracrRNA through a direct repeat sequence to form a dual-guide RNA (dgRNA), or a single RNA transcript fused by a tetraloop to be used a small-guide RNA (sgRNA). The RNA-guided Cas9 causes double-stranded DNA (dsDNA) breaks at the target site that activates the error prone non-homologues end joining (NHEJ) repair pathway resulting in deleterious mutations and subsequent target gene inactivation. As a result of the ease of its application, CRISPR/Cas9 has become a staple in biological research, and is currently being developed for clinical applications (reviewed in^3^).

Several modifications to the sgRNA have been explored in order to improve Cas9 activity. Some of the original CRISPR/Cas9 systems were expressed from Pol III promoters as a fusion sgRNA, resulting in a loss of efficiency due to the shortened upper stem region of the direct repeat^2^. Extending the upper stem with wild-type sequence improved expressed sgRNA genome editing^4^ and the fluorescent signal resolution of Cas9 used in imaging applications^5^. Furthermore, sgRNAs expressed from Pol III expression cassettes were further improved by the disruption of a natural poly-T tract in the tracrRNA, which prevented premature transcription termination^4,5^.

These modifications were utilized with expressed sgRNAs, but sgRNAs can be preloaded into recombinant Cas9 protein and applied as a ribonucleoprotein (RNP) complex, which is preferable as a result of reduce toxicity^6^ and off-targeting^7^. Chemical modification of the crRNA and tracrRNA has also been explored to improve CRISPR/Cas RNP activity in primary cells^8^ and *in vivo*^9^ presumably by enhancing RNA stability. Furthermore, commercial companies have explored length reduction of crRNAs and tracrRNAs to reduce costs and improve Cas9 RNP activity. Although chemical and length modifications have been explored, and, apart from the sequence alterations to improve Pol III expressed sgRNAs, no attention has been given to the modification of tracrRNA nucleotide sequence to improve Cas9 RNP activity.

Here we identify specific sequence modifications to the gRNA that significantly enhance Cas9 RNP activity. Focusing on the replacement of uridines, we screened U-modified tracrRNAs and identified nucleotide substitutions that improved Cas9 RNP knockdown of human immunogenicity virus (HIV) reporter cell lines, and observed this enhancement with several different crRNAs targeting the long terminal repeat (LTR) of HIV. Furthermore, we show that these U-modified tracrRNAs improved knockout activity of a different target gene, specifically that of the essential HIV co-receptor, C-C chemokine receptor type 5 (CCR5), resulting in increased reduction of CCR5 surface receptor and concordant enrichment of indels at the target site. Lastly, using the U-modified tracrRNAs, we observed enhanced indel formation in primary CD4+ T-cells. These data suggest this improvement in Cas9 RNP technology could potentially prove useful for research as well as therapeutic applications.

## Results

### Sequence-modified tracrRNA can improve Cas9 RNP activity

To investigate the importance of uridines within expressed sgRNAs, the uridines were replaced with either Adenines (A), Guanines (G) or deleted. This modification of the tracrRNA was prompted by experiments with *in vitro* transcribed sgRNAs with chemically-modified pyrimidines, which demonstrated that chemically-modified uridines resulted in a loss of Cas9 activity (data not shown), and replacing uridines within the tracrRNA with purines may allow for a functional RNase-resistant *in vitro* transcribed tracrRNA. With this in mind, U-modified sgRNAs were designed and expressed off a Pol III U6 promoter with a target sequence directed to the essential Trans-activation response (TAR) element in the LTR of HIV (Supp. Table 1). HIV was selected as the model target as a result of growing interest to inactive or excise proviral HIV in the host genome as a possible “sterile cure” approach (reviewed in^10^). The sgRNAs were transfected with a Cas9 expression vector into TZM-bl cells, a cell line with an LTR expressing luciferase, and activity was assessed at 48 hours post-transfection. It was noticed that several U-modified sgRNAs demonstrated improved knockdown activity over a unmodified sgRNA (sgRNA-UM), with U-modified sgRNA-8 resulting in ~40% increase in knockdown activity (Supp. Fig. 1A). Those sgRNA target sites that demonstrated improved activity were amplified and subjected to TIDE analysis, which determines the percentage of indels through a decompress algorithm to deconvolute automated sequencing^11^. Notably, there was a general trend to improve indel percentage with several of the sgRNAs, but sgRNA-8 had the highest level of indels with an increase of ~2-fold (Supp. Fig. 1B). Encouraged by these data obtained with expressed DNA vectors, Cas9 RNPs were explored instead for a number of reasons, (a) lengthy expression of CRISPR/Cas could result in accumulation of indels in off-target sites, (b) concerns around random DNA integration of the expression vectors^12^, and (c) recognition of bacterial DNA CpG motifs activating innate immunity^6^. Cas9 delivered to cells as an RNP reduces off-target activity^7^, and does not require DNA components and is quickly emerging as the most precise and effective route to utilize this technology for research and *in vivo* applications.

**Figure 1:**
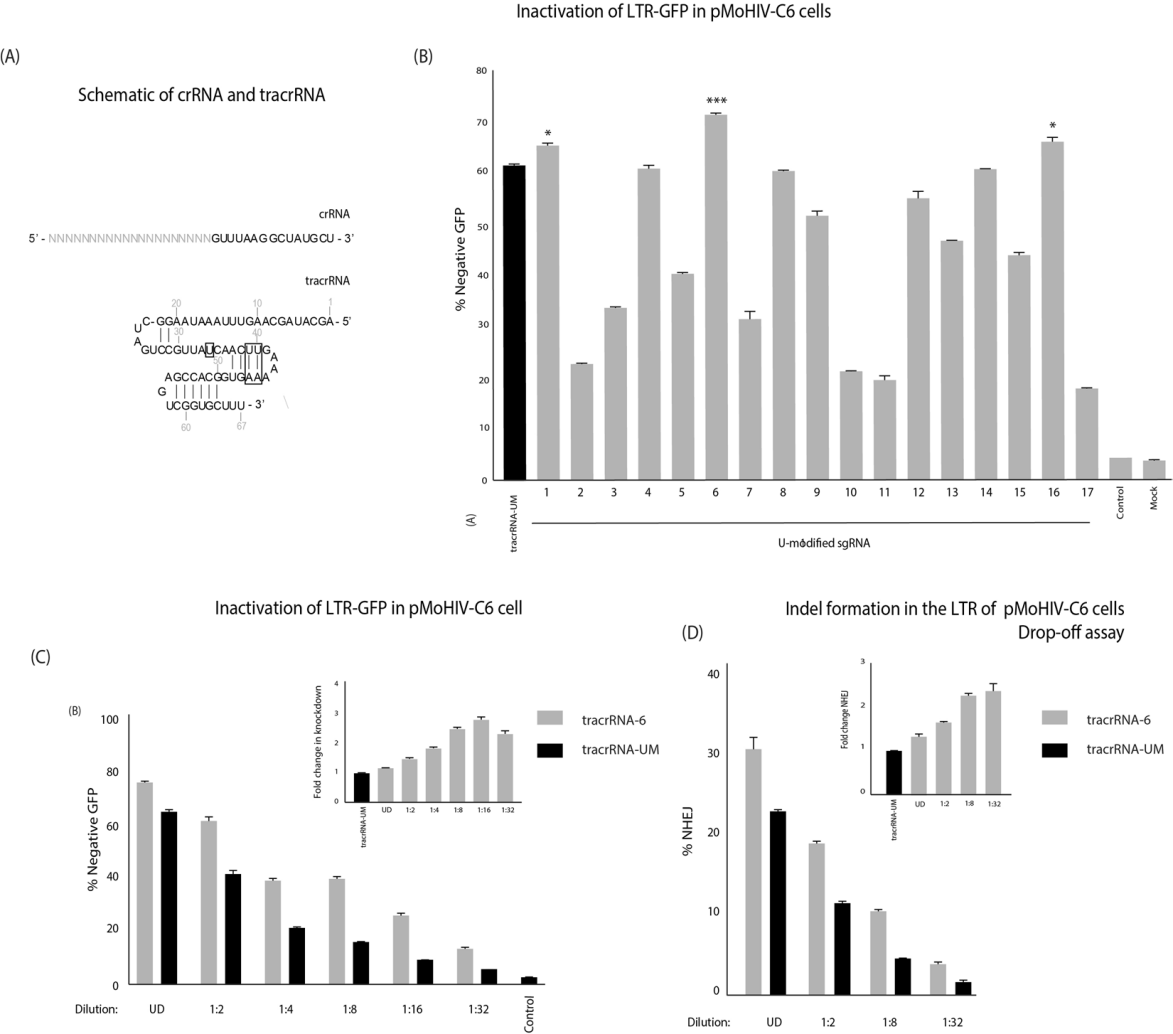
Identification of a tracrRNA with improved Cas9 RNP activity. (**A**) A schematic of the crRNA and tracrRNA. The target sequence is represented by N(20) in the crRNA. The boxed nucleotides are altered in tracrRNA-6 and 19. (**B**) A series of U-modified tracrRNAs (1–17) were annealed with a TAR6 crRNA and transfected into pMoHIV-C6 cells. GFP expression was assessed by FACS at 48 hours post-transfection. An unmodified tracrRNA-(tracrRNA-UM) was included as a comparative control. Untransfected cells (Mock) or a transfection without a dgRNA (control) were included as negative controls. (**C**) A serial dilution of the tracrRNA-6 and tracrRNA-UM were transfected into pMoHIV-C6 cells, and the levels of GFP were assessed by FACS. The embedded image reflects fold change in knock-down activity relative to the tracrRNA-UM at each dilution. (**D**) Total DNA was extracted 48 hours post-transfection and the percentage of indels was determined by drop-off assay using ddPCR. The embedded image reflects fold change in indels relative to the tracrRNA-UM at each dilution of the tracrRNA-6. The errors bars represent standard error of the mean (SEM) of duplicate treated samples and experiments performed in duplicate, and the experiment was repeated twice.*p < 0.05, ***p < 0.001 were obtained by one-way ANOVA and Dunnett’s test.

A panel of tracrRNAs were generated through *in vitro* transcription with U-modified sequences and annealed with an anti-TAR crRNA to form a dual-guide RNA (dgRNA) (Supp. Table 2). This “2-part” system, using a separate CRISPR-RNA (crRNA) and tracrRNA, was selected for investigation as a result of its facile modularity (Fig. 1A). These dgRNAs were preloaded into a Cas9 RNP complex, and transfected into a pMoHIV clone 6 cell line (pMoHIV-C6), a clonal HEK293 cell line with a LTR driving high levels of GFP expression (data not shown). Forty-eight hours post-transfection the levels of GFP were determined by FACS. Three of the U-modified tracrRNAs demonstrated a higher percentage of GFP negative cells, namely U-modified tracrRNA-1, 6 and 16,compared to the unmodified control, tracrRNA-UM (Fig. 1B). Interestingly, both tracrRNA-6 and 16 had Us replaced in the ‘linker’ region of the tracrRNA. The tracrRNA-6, containing a U34A change (Fig. 1A, Supp. Table 2),demonstrated the most pronounced increase in activity and was selected for further investigation. The Cas9 RNP with tracrRNA-6 was serially diluted and consistently exhibited higher levels of GFP knockdown. Importantly, at a 1:2 dilution, the tracrRNA-6 knockdown was comparable to undiluted transfection of RNP with tracrRNA-UM (Fig. 1C). At lower dilutions (1:4, 1:8, 1:16 and 1:32), the knockdown percentage was approximately double that of the tracrRNA-UM (Fig. 1C, embedded image). To assess whether the tracrRNA-6 improved indel formation, the target site in the LTR was assessed by a drop-off assay, which measures indel formation using droplet-digital PCR (ddPCR) through the loss of probe binding to the mutated target site^13^. The results from this drop-off assay matched the knockdown data, as the tracrRNA-6 demonstrated higher levels of indel formation compared to tracrRNA-UM, and were more pronounced at lower dilutions (Fig. 1D). To determine if the types of mutations generated for tracrRNA-6 were different compared to tracrRNA-UM, the target site was subject to TIDE analysis. The levels of indel formation observed by TIDE corroborated the drop-off assay (Supp. Fig. 2A), and the types of mutations were similar across both groups, although higher levels of targeted mutations were observed in the tracrRNA-6 treated cells compared to the unmodified control (Supp. Fig. 2B).

### Additional sequence-modifications to the tracrRNA further improve Cas9 RNP activity

**Figure 2:**
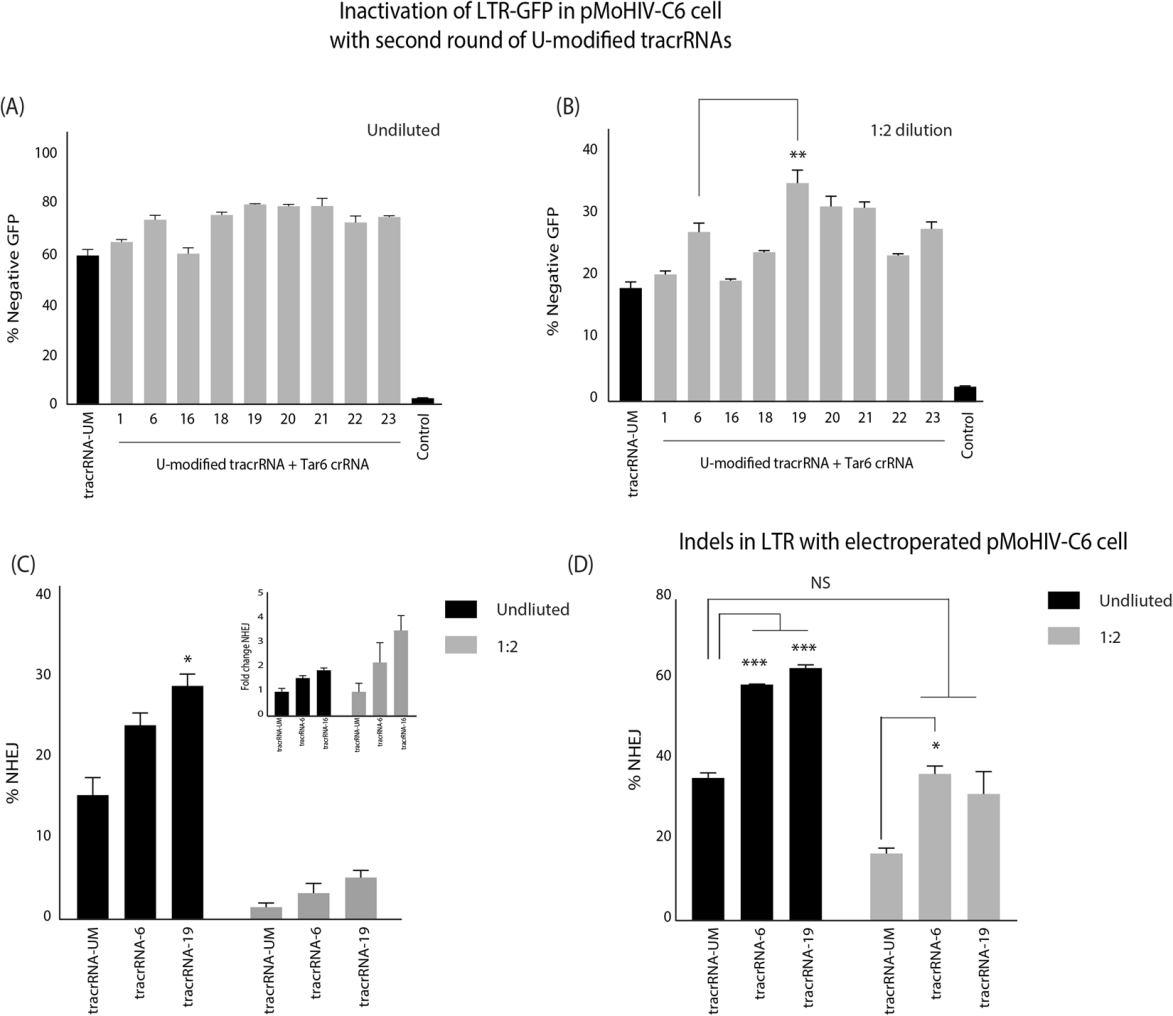
Secondary screen of tracrRNAs with improved Cas9 RNP activity. A second series of U-modified tracrRNAs (18–23) were annealed with a TAR6 crRNA and the dgRNA transfected into pMoHIV-C6 cells either (**A**) undiluted or (**B**) at a 1:2 dilution.GFP expression was determined by FACS. A transfection without sgRNA (control) was included as a negative control. **(C)** Total DNA was extracted and the percentage of indels was determined by drop-off assay using ddPCR. The embedded image reflects fold change in indels relative to the tracrRNA-UM at each dilution. **(D)** The pMoHIV-C6 cells were electroporated with tracrRNA-UM, tracrRNA-6, or tracrRNA-19 annealed to TAR6 crRNA, and the percentage of indels was determined by drop-off analysis. The errors bars represent standard error of the mean (SEM) of samples treated in duplicate, and the experiment was repeated twice. *P < 0.05, **p < 0.005, ***p < 0.001 were obtained by one-way ANOVA and Dunnett’s test.

To determine if the activity of Cas9 RNP could be improved further through additional tracrRNA sequence changes, the tracrRNA-6 modification (U34A) was combined with the changes in tracrRNA-1 (U13A) and tracrRNA-16 (U32G) (Fig. 1A, Supp. Table 2). Furthermore, we have observed that stabilizing stemloop-2 in the tracrRNA by replacing the U-Ts with G-Cs, rescued Cas9 RNP activity in tracrRNAs with deleterious sequence changes (data not shown), and was also included in the screen (Supp. Table 2). The Cas9 RNPs loaded with dgRNAs containing the new U-modified tracrRNAs were transfected into pMoHIV-C6 cells either undiluted or at a 1:2 dilution, and levels of GFP were assessed 48 hours post-transfection. With the undiluted transfection, there was several tracrRNAs that had slight, but not significant, improvement of activity over the tracrRNA-6 (Fig. 2A), but at 1:2 dilution tracrRNA-19 demonstrated a statistically significant increase in GFP knockdown (Fig. 2B). The tracrRNA-19 contained U34A and stemloop-2 stabilizing modifications (Fig. 1A, Supp. Table 2). The analysis of indels by drop-off assay demonstrated that mutations at the TAR target site was slightly higher with tracrRNA-19 than tracrRNA-6 (28% versus 23%), and ~2-fold more than tracrRNA-UM (28% versus 15%) (Fig. 2C). Again, at the lower dilutions, the fold change in indels became more pronounced with tracrRNA-6 and tracrRNA-19 (Fig. 2C, embedded image).

To ensure the effects where not dependent on lipofection-mediated delivery of RNPs, Cas9 RNP dgRNAs were electroporated into pMoHIV-C6 cells, and the levels of indel formation was again ~2-fold higher with the U-modified tracrRNAs than the tracrRNA-UM (Fig. 2D). Importantly, at the 1:2 dilution, the U-modified tracrRNAs demonstrated a similar level of indel formation to the undiluted tracrRNA-UM.

### Sequence-modified tracrRNA can improve Cas9 RNP activity of alternative crRNAs

**Figure 3:**
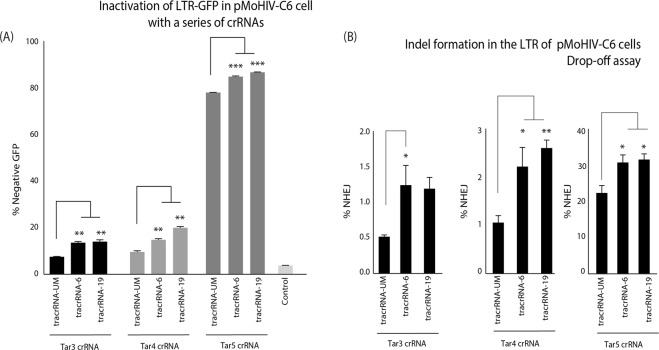
Modified tracrRNAs improve Cas9 RNP activity at other TAR crRNAs sites. The tracrRNA-UM, tracrRNA-6 and tracrRNA-19 were annealed with a TAR3,4 and 5 crRNAs and transfected into pMoHIV-C6 cells.**(A)** The levels of GFP expression was assessed by FACS, and (**B**) indel formation measured by a drop-off assay. A transfection without dgRNA (control) was included as negative control. The errors bars represent standard error of the mean (SEM) of samples treated in duplicate, and the experiment was repeated twice.*p < 0.05,**p < 0.005, ***p < 0.001 were obtained by one-way ANOVA and Dunnett’s test.

**Figure 4:**
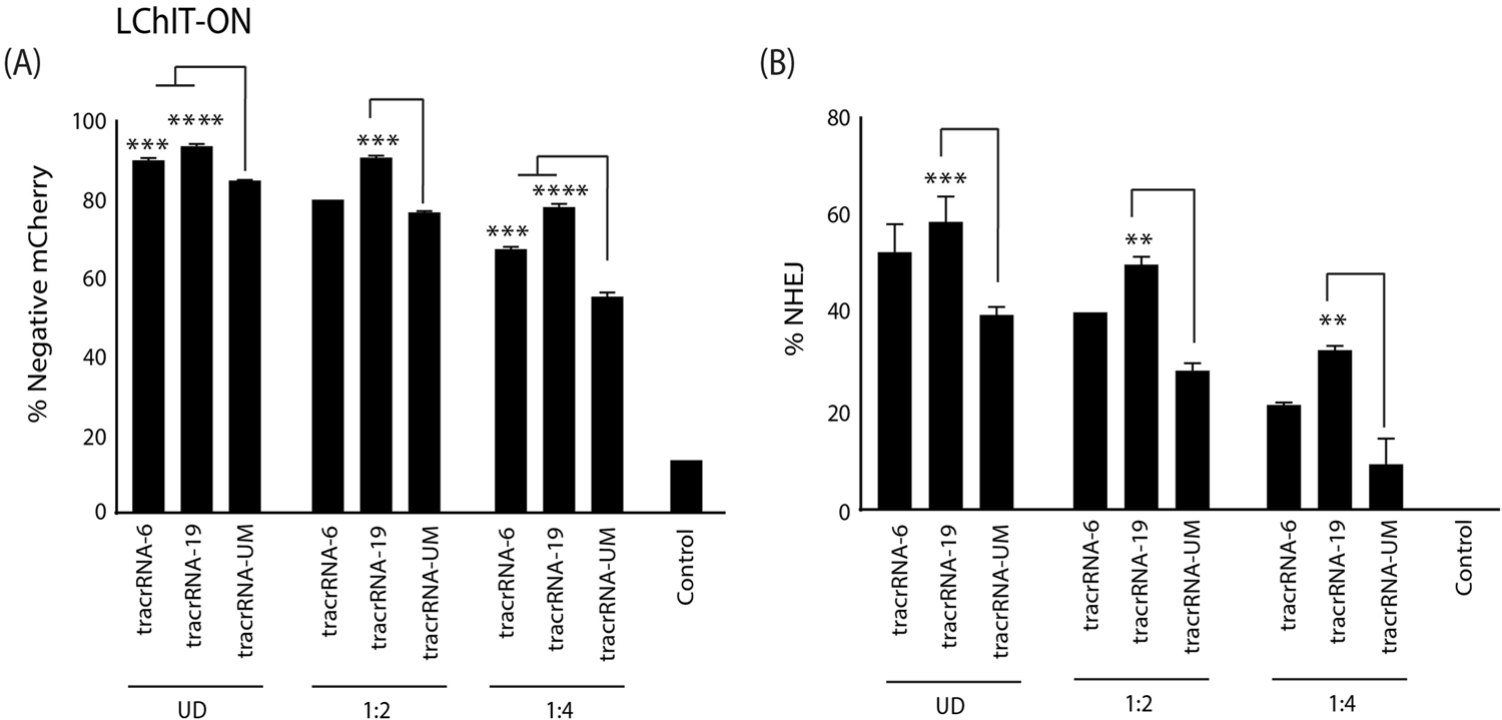
U-modified tracrRNAs improve activity in a LTR model with Tat: The tracrRNA-UM, tracrRNA-6 and tracrRNA-19 were annealed with a TAR6 crRNA electroporated into LChIT-ON cells. **(A)** The level of mCherry expression was assessed by FACS and **(B)** indel formation was measured by a drop-off assay at 72 hrs post- electroporation. The errors bars represent standard error of the mean (SEM) of samples treated in triplicate. **p < 0.005, ***p < 0.001, p < 0.0005 **** were obtained by one-way ANOVA and Dunnett’s test.

To validate that this improvement in Cas9 RNP activity was not crRNA specific, three other crRNAs targeting the TAR loop were tested (Supp. Table 3). The crRNAs for TAR3, 4 and 5 were annealed with a tracrRNA-UM, tracrRNA-6 or tracrRNA-19, and Cas9 RNPs were transfected into the pMoHIV-C6 cells. The percentage of GFP negative cells was increased for all three crRNAs with U-modified tracrRNAs compared to tracrRNA-UM (Fig. 3A), and improvement in indel formation was verified by drop-off assay (Fig. 3B). However, tracrRNA-19 only showed a slight improvement in activity with TAR4 (similar to TAR6), and overall did not significantly improve knockdown or indels relative to TAR3 or TAR5, which suggests some target specific effects between tracrRNA-6 and tracrRNA-19. Collectively, these data demonstrate that the modified tracrRNAs can significantly improve Cas9 RNP activity with other crRNAs compared to an unmodified tracrRNA. Finally, we wanted to determine if the modified tracrRNA could have an effect in a different LTR model, which has the presence of Tat regulating the LTR. The LChIT cell line was used that has a Tat positive feedback loop driving high levels of mCherry expression^14^, and TAR sgRNAs have shown anti-HIV activity in infectious models of HIV^15^. The LChIT cells were electroporated with TAR6 crRNA annealed to the modified tracrRNA-6 and 19 and showed improved knockdown of mCherry expression **(**Fig. 4A**)** and indel formation **(**Fig. 4B**)** across a range of dilutions. These data suggest that the modified tracrRNA would have activity in various LTR models and verifies the improved activity in a different cell line.

### Sequence-modified tracrRNA can improve Cas9 RNP activity in CD4+ T cells

**Figure 5:**
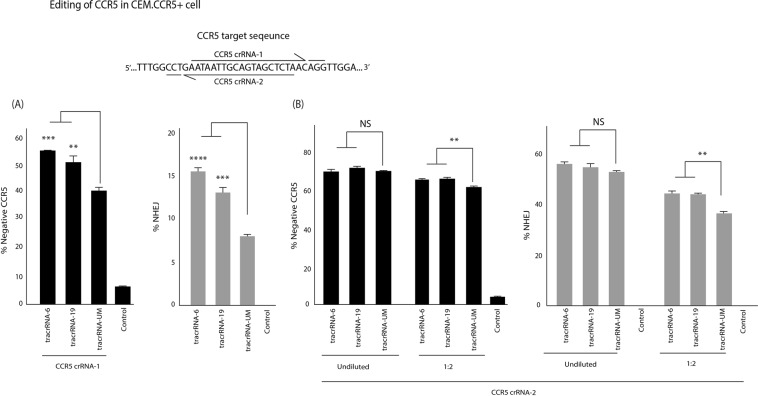
The modified tracrRNAs improved CCR5 knockdown. The tracrRNA-UM, tracrRNA-6 and tracrRNA-19 were annealed with a **(A)** CCR5-crRNA 1 and **(B)** CCR5-crRNA 2 and electroporated into CEM.CCR5+ cells. The level of CCR5 expression was assessed by FACS (black bars) and indel formation was measured by a drop-off assay (grey bars). The errors bars represent standard error of the mean (SEM) of samples treated in triplicate, and the experiment was repeated twice. **p < 0.005, ***p < 0.001, p < 0.0005 ****were obtained by one-way ANOVA and Dunnett’s test.

The enhancement of Cas9 RNP activity was observed in cell lines and targeted to HIV. To determine whether the tracrRNA could improve activity in other cell lines and a different target site the CCR5 co-receptor, essential for R5-tropic HIV-1 entry, was assessed. Notably, inactivation of CCR5 is currently being used to generate HIV-resistant CD4+ T-cells^16^ in a phase I clinical setting^17^. Two target sites were selected within the CCR5 gene and the crRNAs where annealed with tracrRNA-6 and 19 and loaded into Cas9 RNPs that were electroporated into a T-cell line engineered to express CCR5, CEM.CCR5. Interestingly, target site 1 (CCR5-1 crRNA) was significantly improved with the U-modified tracRNAs (tracrRNA-6 > tracrRNA-19) as determined by the reduction in CCR5 surface expression and improved indel formation at the target site (Fig. 5A) and an improvement in indels was observed at a 1:2 dilution (Supp. Fig. 3A). The second target site didn’t demonstrate a notable improvement in the undiluted samples, but showed a significant difference in CCR5 surface reduction and indel formation at 1:2 and 1:4 dilution compared with a tracrRNA-UM (Fig. 5B and Supp. Fig. 4).

**Figure 6:**
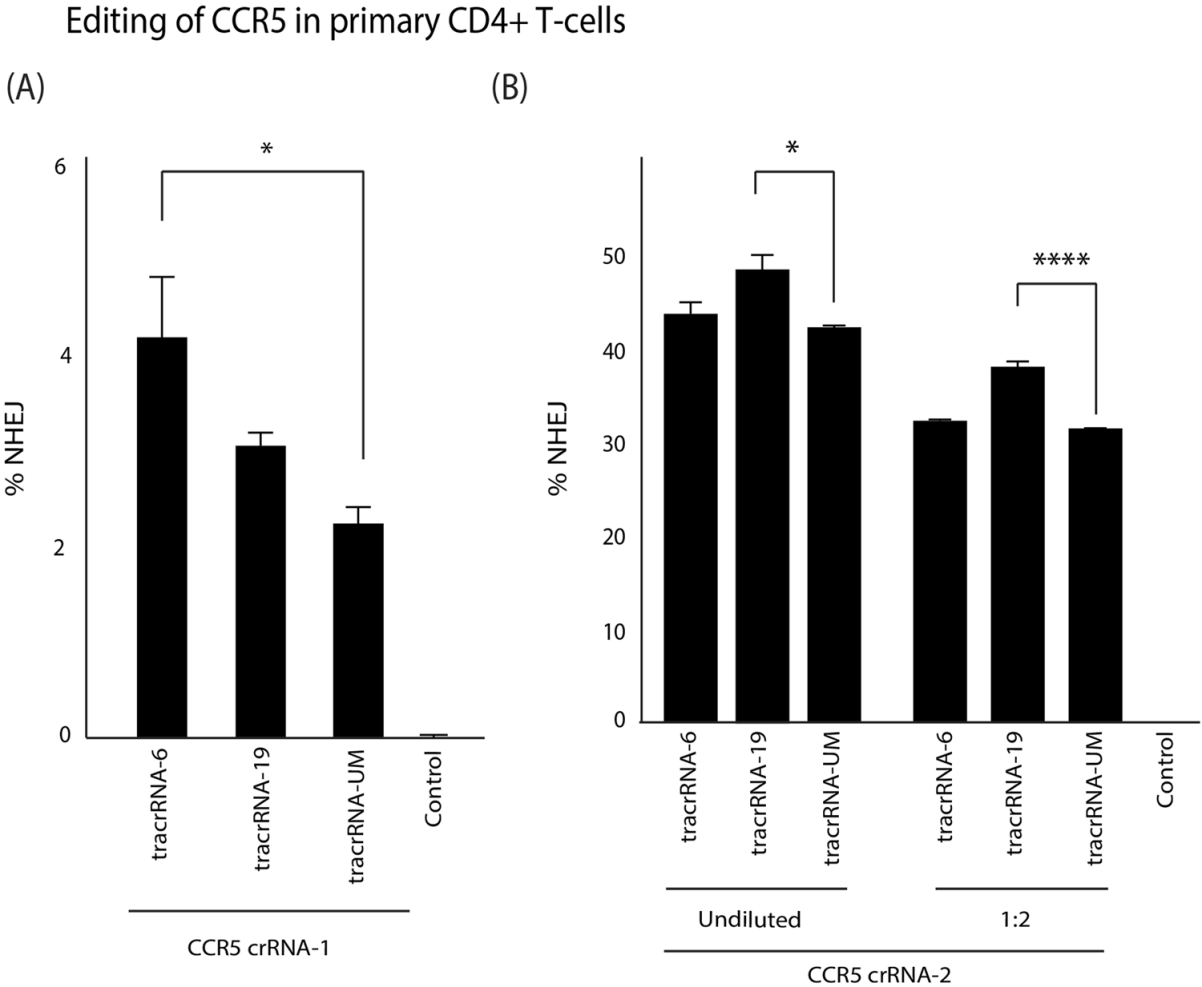
Modified tracrRNAs improve Cas9 RNP indel formation in CD4+ T-cells. The tracrRNA-UM, tracrRNA-6 and tracrRNA-19 were annealed with a **(A)** CCR5 crRNA-1 and **(B)** CCR5 crRNA-2 and electroporated into purified and activated CD4+ T-cells. CCR5-2 was transfected undiluted or at 1:2 dilution. The levels of indel formation was measured using a drop-off assay. The errors bars represent standard error of the mean (SEM) of samples treated in triplicate. *p < 0.05, ****p < 0.0005 were obtained by one-way ANOVA and Dunnett’s test.

To verify that these observations in primary cells, activated CD4+ T-cells were isolated and purified from healthy donors and electroporated with Cas9 RNP preloaded with U-replaced tracrRNA and the percentage of indels was measured at 72 hours post-treatment. A significant improvement of indel formation with CCR5-1 crRNA annealed to tracrRNA-6 was observed (Fig. 6A) and improved indels were also observed at a 1:2 dilution with both tracrRNA-6 and tracrRNA-19 (Supp. Fig. 3B). Interestingly, with CCR5-2 crRNA, there was no observable improvement in indel formation with tracrRNA-6 in CD4+ T-cells (Fig. 6B). However, as observed previously, tracrRNA-19 demonstrated a significant increase in indel formation and, importantly, the levels of indels at a 1:2 dilution was comparable to that of a undiluted tracrRNA-UM (Fig. 6B). The indel activity of CCR5-2 with modified tracrRNAs was further validated in HEK293 cells that showed the same pattern of activity as CD4+ T-cells **(**Fig. S5).

### Sequence-modified tracrRNAs improve Cas9 RNP activity at additional target sites

**Figure 7:**
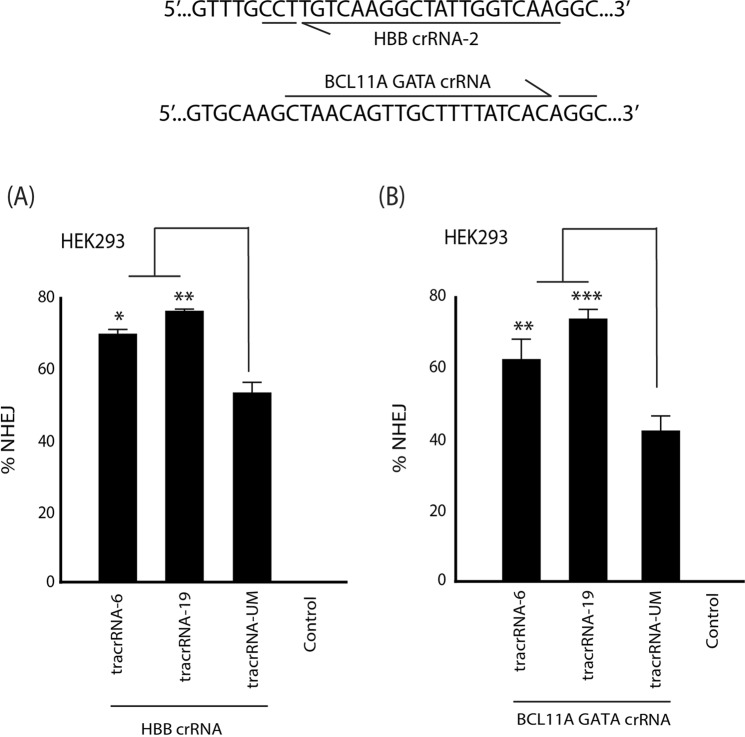
Modified tracrRNAs enhanced indels at additional target sites. HEK293 cells were electroporated with tracrRNA-UM, tracrRNA-6, or tracrRNA-19 annealed to a crRNA targeting **(A)** HBB or **(B)** BCL11A GATA site, and the percentage of indels were determined using a TIDE assay. The errors bars represent standard error of the mean (SEM) of samples treated in triplicate, and the experiment was repeated twice. *P < 0.05, **p < 0.005, ***p < 0.001 were obtained by one-way ANOVA and Dunnett’s test.

Moving beyond HIV, we wanted to determine if the modified tracrRNAs could improve activity at alternative targets site. We choose two additional sites that are of significant interest in correcting sickle cell disease (SCD) through the activation of fetal hemoglobin by targeting the BCL11A repressor motif in the HBB locus^18^ or GATA signal for erythroid-specific repression of BCL11A^19^. HEK293 cells were electroporated with the modified tracrRNA annealed to crRNA targeting HBB **(**Fig. 7A**)** and BCL11A GATA **(**Fig. 7B**)** and indels were quantified 72 hrs later by TIDE analysis. The level of indels was significantly improved at the HBB and BCL11A target sites with a ~32% and 23% improvement with tracrRNA-19, respectively.

## Discussion

We report here that particular nucleotide changes, specifically located in the “linker” region (34A) and stem-loop 1 (U39G-U40G) in the tracrRNA can significantly improve Cas9 RNP activity. Apart from the disruption of the Poly-T tract or length changes to an sgRNA^4,5^, this is the first comprehensive exploration of sequence changes to the tracrRNA to improve Cas9 RNP activity. These data suggest that additional sequence changes could be explored, beyond the changes in this study, that may further improve activity. Whether the modifications will improve sgRNAs, and not only dgRNA activity, will also be the focus of future study.

Although it is clear there is an increase in activity with the U-modified tracrRNAs used in this study, there was variation in activity with specific crRNAs in different cells (Figs 5B and 6B: CCR5-2 with tracrRNA-6) as well as differences between crRNAs with modest (TAR5 crRNA: Fig. 3B or CCR5-2: Figs 5B and 6B), or substantial (TAR6: Figs 2 and 4. CCR5-1 Fig. 5A, HBB and BCL11A GATA: Fig. 7) increases in indel formation. Further validation is needed to better understand the circumstances that give rise to improve activity to determine applications that will benefit from using modified tracrRNAs. The TAR6 crRNA showed significant improvements in indel formation within the LTR **(**Figs 2 and 4**)**. A panel of anti-TAR sgRNAs were tested on LChIT models, and the effects translated into effective inhibition of HIV in infectious models^15^, suggesting the improvements observed with modified tracrRNAs would translate into enhanced anti-HIV activity, however, further validation within infectious models will be required to confirm these effects. Furthermore, additional studies are needed in a range of primary tissues such as hematopoietic stem cells (HSC), as well as validate the effects *in vivo*.

The reason these changes improve CRISPR/Cas activity is unknown. The Cas9:sgRNA structure bound to target DNA has elucidated the confirmation of an active catalytic Cas9 complex, and revealed the role of nucleotides that directly interact with Cas9^20^. The U-modified tracrRNAs may alter how Cas9 interacts with dgRNA by improving affinity for the tracrRNA. A sgRNA bound to Cas9 contends with non-specific endogenous RNA, and truncated tracrRNAs that lose essential Cas9 interactions are out competed by non-specific RNA resulting in reduced kinetics^21^ and actvity^22^ probably as a result of reduced affinity for the sgRNA. However, the changes observed here with our modified tracrRNA do not interact with Cas9 RNP and probably do not directly improve Cas9’s affinity for the tracrRNAs. However, this does not preclude that the modifications my fold the tracrRNA into a three dimensional structure that is better recognized by the Cas9 protein.

Alternatively, conformational changes and kinetics may be augmented in the modified tracrRNAs. Impressive research has investigated the energy requirements for Cas9 catalytically activity to overcome mismatches at the target site, and has provided a better understanding of on-targeting versus off-target bias^23^, as well as comprehensive investigations into the functional importance of different modular structures within the sgRNA through sequence changes and truncations^24^. Nevertheless, there are no studies to date that have focused on the sequence composition of tracrRNAs and its contribution to the kinetics in Cas9 conformations during activation. As the Cas9 protein goes through conformation changes, the sequence composition of the tracrRNA may affect its plasticity and result in steric energy barriers. A better understanding of how nucleotide composition contributes to tracrRNA conformational kinetics could help elucidate the impact of the tracrRNA modifications on Cas9 activity. Furthermore, insight into those sequence compositions that effect Cas9 activity could allow for the rational design of tracrRNAs with improved function, and, it is tempting to speculate, that a novel tracrRNA could be artificially evolved under selective conditions that give rise to a tracrRNA with kinetics that favor a more active catalytic Cas9 RNP complex. Nevertheless, further work will be required to determine the reason for the improved Cas9 activity with dgRNAs using U-modified tracrRNAs.

Furthermore, research studies have focused on chemically-modified dgRNAs for *in vivo* applications as a result of the technical and financial constraints synthesizing longer sgRNAs^9^, highlighting the need for approaches that improve dgRNA activity. The wide-spread application of CRISPR/Cas9 has spurred significant effort into the screening of expressed sgRNA target sites *in silico* to identify the composition of ‘highly active’ target sequences^25,26^. Chemical modifications are also used to boost RNA stability and Cas9 RNP activity^8^, but can significantly increase the cost of the RNA synthesis. Nevertheless, a combination of sequence modifications with chemically-stabilized dgRNAs as well as optimized *in silico* targets may enhance Cas9 RNP activity. Therefore, the identification of enhancing sequence changes to the tracrRNA to improve Cas9 RNP activity may represents a cost effective, broad, and easily applicable change to improve current Cas9 RNP technology for various research and *in vivo* applications using dgRNAs.

CRISPR/Cas has been extensively applied to basic research and model development, and higher Cas9 activity would improve knockout signal in experimentation, as well as reduce labor in clonal screens. In therapeutic applications, CRISPR/Cas9 has been used to knockout CCR5 in CD4+ T-cells^27^ and hematopoietic stems cells (HPSC)^16^ and has shown to protect against R5-tropic infection as a possible “functional” cure approach to HIV. However, populations of re-infused unedited or heterozygous edited cells can act as a substrate for HIV replication, and therefore improving the quality of knockout in the reinfusion product may enhance protection from HIV infection. CRISPR/Cas9 has also been applied to eliminate HIV proviral DNA in CD4+ T-cells^28^ and the potent eradication of HIV would benefit from improved Cas9 RNP activity. Alternatively, gene-editing approaches to Sickle Cell Anemia that relies on the activation of fetal hemoglobin by modification of globin locus in HPSC stem cells^29^ or mutation of erythoid-specific enhancer elements^19^ correlates with the levels of gene-editing. Furthermore, the potency of the Cas9 RNP complex was vital to obtaining effective editing *in vivo* using non-viral delivery systems^9^. Finally, we find here that a reduced amount of Cas9:dgRNA could result in comparable levels of indel formation when compared to that of the unmodified tracrRNA (Figs 1D, 3, 5A and 6B). The generation of clinical GMP grade components is expensive, and the ability to utilize less reagent would significantly reduce the cost of therapeutic applications. The improvement in Cas9 RNP activity through the development of U-modified tracrRNA approaches using dgRNAs may be able reduce labor costs and enhance gene knockout for both research and clinical applications.

## Materials and Methods

### Cell lines

The pMoHIV cell line was generated as described in Shrivastava *et al*.^30^. The cells were then clonally expanded by diluting to 0.5 cells/well in a 96-well plate and a pMoHIV clone 6 (pMoHIV-C6) was identified with high levels of GFP expression (data not shown). The generation of LChIT cells has been previously described^14^. The CEM.NKR CCR5+ (referred to as CEM.CCR5+) was obtained through the NIH AIDS Reagent Program, Division of AIDS, NIAID deposited by Dr Alexandra Trkola^31–33^. For the Primary CD4+ T-cells, leukapheresis products were obtained from healthy donor under protocols approved by the City of Hope Institutional Review Board. PBMCs were isolated by density gradient centrifugation over Histopaque (Sigma, MO, USA) and CD4+ T-cells were isolated with CD4 negative selection EasySep™Cell Separation Kit (STEMCELL, Vancouver, Canada). CD4+ T cells were activated using ImmunoCult™ Human CD3/CD28 T cell activator for 3 days prior to electroporation with Cas9 RNPs.

### Cell culture

The pMoHIV-C6 cells were cultured in Dulbecco’s modified Eagle’s medium (Thermo Fisher Scientific, MA, USA) supplemented with 10% fetal bovine serum (Thermo Fisher Scientific, MA, USA). The CEM.CCR5+ cells were maintained in RPMI 1640 (Thermo Fisher Scientific, MA, USA) supplemented with 10% fetal bovine serum. The CD4+ T-cells were maintained in RPMI + 10% FBS with 50 U/ml of IL-2. All cells were incubated at 37 °C and 5% CO^2^.

### Generation of tracrRNAs

The templates for tracrRNA transcription were ordered as complementary oligomers (250 µmole scale, PAGE purified: IDT, CA, USA) and contained a T7 promoter at the 5′ end of the tracrRNA sequence (T7 promoter sequence: 5′-TAATACGACTCACTATAGG-3′). The oligomers were mixed at an equal concentration at 500 ng/µl and heated to 95 °C for 5 min and slow cooled to room temperature. The tracrRNAs were *in vitro* transcribed from the templates using the DurasScribe® kit according to the manufacturer instructions (Lucigen, WI, USA) but unmodified ribose nucleotides (NTPs) were used in the reaction (NEB, MA, UK). The reaction was cleaned up using the RNA Clean and Concentrator-25 kit (Zymo Research Corporation, CA, USA) according to the manufacturer instructions, eluted in 100 µl of RNAse-free water and precipitated with 10 µl of 3 M Sodium Acetate and 250 µl of 100% Ethanol overnight at −20 °C. The reaction was centrifuged at 4 °C for 30 min, the pellet washed with 70% ethanol and resuspended in 20 µl of duplex buffer (IDT, CA, USA) and stored at −80 °C until ready to use. The crRNAs were ordered as HPLC-purified RNA oligomers (IDT, CA, USA), resuspend in duplex buffer at 100 µM concentration and stored at −80 °C until needed.

### Cas9 RNP transfection and electroporation

For transfection of Cas9 RNPs, the crRNA and tracrRNAs were diluted to 6 µM in duplex buffer (IDT, CA, USA) and mixed at equal volumes. The crRNA and tracrRNA were annealed by heating to 95 °C for 5 min and slow-cooled to room temperature to form the dgRNA. The Cas9 RNP complex was formed by mixing 0.5 µl of 3 µM Alt-R® S.p.Cas9 nuclease V3 (IDT, CA, USA) with 0.5 µl of 3 µM of sgRNA and made up to 55 µl in cytoplasmic-like buffer (120 mM KCl, 0.15 mM CaCl_2_, 10 mM KH_2_PO_4_ (pH 7.6), 2 mM EGTA (pH 7.6), 5 mM MgCl_2_) with 100 µg/ml BSA (NEB, MA, UK) and incubated at room temperature for 15 min. Fifty-five microliters of OptiMEM with 1.5 µl RNAiMAX Reagent (Thermo Fisher Scientific, MA, USA) was added to the RNP mixture and incubated for 20 min at room temperature. A total of 50 µl was added to each well of a 48-well plate and then 200 µl of 6.4 × 10^5 cells/ml of pMoHIV-C6 cells was added. For the electroporation of Cas9 RNPs, the sgRNA and Cas9 were diluted to a 20 µM concentration and 0.6 µl of each was mixed with Buffer R to a total volume of 7 µl. The reaction was incubated for 15 min at room temperature and then 5 µl mixed with 100,000 cells. The reaction was electroporated using the 10 µl Neon® transfection system with the following settings for the pMoHIV-C6 cells: 1700 V, 20 ms, 1 pulse, for the CEM.CCR5+ and LChIT cells: 1230 V, 40 ms, 1 pulse, and for the CD4+ T-cells: 1600 V, 10 ms, 3 pulse. The cells were added to 500 ul of pre-warmed media in a 48-well plate, and pMoHIV-C6 and CEM.CCR5/CD4+ T-cells were processed for further analysis at 48 hrs and 72 hrs post-transfection, respectively. The reaction mixtures were diluted in the Cytoplasmic-like buffer or Buffer R to make up the lower dilution reactions.

### FACS analysis

To detect the levels of GFP in the pMoHIV-C6 cells, the cells were trypsinized (0.25% Trypsin-EDTA, Gibco, Thermo Fisher Scientific, MA, USA), and washed with 1x DPBS (Corning, NY, USA). A total of 10,000 events was collect on single cells using the BD Accuri^TM^ C6, and analyzed by FlowJo vX3.05470 software. Detection of mCherry in LChIT cells has been previously described^14^. For the detection, of CCR5, CEM.CCR5+ cells were centrifuged at 1000 rpm for 5 min and resuspened in 100 µlof PBS + 1% bovine serum albumin (BSA) with 5 µl of a Mouse APC anti-CCR5 (Cat. no 556903, BD Bioscience, CA, USA) for 30 min at room temperture in the dark. To wash, 1 ml of PBS + 1% BSA was added to the tube and centrifuged at 1000 rpm for 5 min, and the pellet was resuspended in PBS + 1% BSA. A total of 10,000 events was collect on single cells using the BD Accuri^TM^ C6, and analyzed by FlowJo vX3.05470 software.

### Quantification of indels

To determine the level of indel formation, DNA was extracted from the pMoHIV-C6 or CEM.CCR5+ cells and either TIDE analysis or a drop-off assay was performed. For TIDE analysis, the target site in the LTR was PCR amplified using the KAPA2G Fast HotStart ReadyMix PCR Kit (Roche, Basel, Switzerland) according the manufacturer’s instructions with LTR-F and R primers (Supp. Table 4) with the following PCR conditions: Initial denaturation for 95 °C for 3 min, then 34 cycles with a 95 °C denaturation for 15 sec, 60 °C annealing for 15 sec, and 72 °C extension for 15 sec, and final extension at 72 °C for 2 min. The PCR products were confirmed by agarose gel electrophoresis and automated sequencing was performed with the same primer sets. The chromatograms were analyzed using the TIDE webtool^11^.

The drop-off assay was performed as described elsewhere^13^. Briefly, 50 ng of total genomic DNA was mixed with ddPCR™ supermix for probes (No dUTP), and the target site was amplified with primers specific for the edited site with a FAM-conjugated target probe and HEX-conjugated reference probe (Supp. Table 4). Droplets were generated using the QX200™ AutoDG™ Droplet Digital™ PCR system. The droplets were sealed in a 96-well plate and the DNA was amplified with the following conditions: initial denaturation at 95 °C for 10 min, then 40 cycles with a 94°C denaturation for 30 seconds, 55°C annealing for 15 seconds and 72°C extension for 1 min with a final incubation at 98 °C for 10 min. The droplets were analyzed on a QX200™ droplet reader using QuantaSoft™ software. The percentage of NHEJ was determined as the number of [HEXposFAMneg/(HEXposFAMneg + HEXposFAMpos)] × 100.

### Institutional approvals

All human cells and manipulation of these cells was carried out in accordance with City of Hope Institutional Biosafety Committee (IBC) following IBC 16006 protocol issued to the Morris laboratory. All methods were carried out with the highest standard and in accordance with the relevant guidelines and regulations imposed at the City of Hope. All donors of human T cells were 18 years or older, and informed consent and de-identification of the donor information was carried out in accordance with the principals and guidelines imposed at the City of Hope.

## Supplementary information

Supplemental materials
