# Supplementary material for: Improved Cas9 activity by specific modifications of the tracrRNA
Source: Sci Rep. 2019 Nov 6;9:16104. doi: 10.1038/s41598-019-52616-5 (PMC6834579; doi:10.1038/s41598-019-52616-5)
Supplement: Supplementary file 1 — Supplemental materials [file 41598_2019_52616_MOESM1_ESM.docx]

**Improved Cas9 activity by specific modifications of the tracrRNA**

Tristan Scott^1^, Ryan Urak^1^, Citra Soemardy^1^, and Kevin V. Morris ^1,*^

^1^Center for Gene Therapy, City of Hope – Beckman Research Institute and Hematological Malignancy and Stem Cell Transplantation Institute at the City of Hope. 1500 E. Duarte Rd., Duarte, CA, 91010, USA

^*^Corresponding author: kmorris@coh.org

Key words: CRISPR/Cas, Human immuno-deficiency virus (HIV), CCR5, modified tracrRNA, dual-guide RNAs

**SUPP. MATERIALS AND METHODS**

**Construction of the U6-sgRNAs**

To generate the U6-exprssed sgRNAs, the sgRNAs were amplified from U6 Pol III with a U6-F primer and with a reverse Ultramer® (IDT, CA, USA) overlapping with 3' end of the U6 promoter and included the TAR6 target with the tracrRNAs (Supp. Table 1). The sgRNAs were amplified using KAPA2G Fast HotStart ReadyMix PCR Kit (Roche, Basel, Switzerland) and ligated into the InsTA PCR cloning kit (Thermo fisher scientific, MA, USA). The U6-sgRNAs were confirmed by automated sequencing.

**Assessing activity of U6-expressed U-modified sgRNAs:**

To assess the levels of activity of the U-modified sgRNAs, TZM-bl cells were transfected in triplicate using Lipofectamine 3000® (Thermo fisher scientific, MA, USA) with the U-modified sgRNAs and a Cas9 expression vector (px458, Addgene #48138). A vector expressing *Renilla* luciferase was included as a background control (pRL-CMV, Promega, WI, USA). At 48hrs post-transfection, a Dual-luciferase®Reporter Assay was performed according to manufacturer's instructions and luciferase activity detected on the Glomax® Explorer system (Promega, WI, USA). The levels of Firefly luciferase were normalized to *Renilla* luciferase, and made relative to the unmodified control.


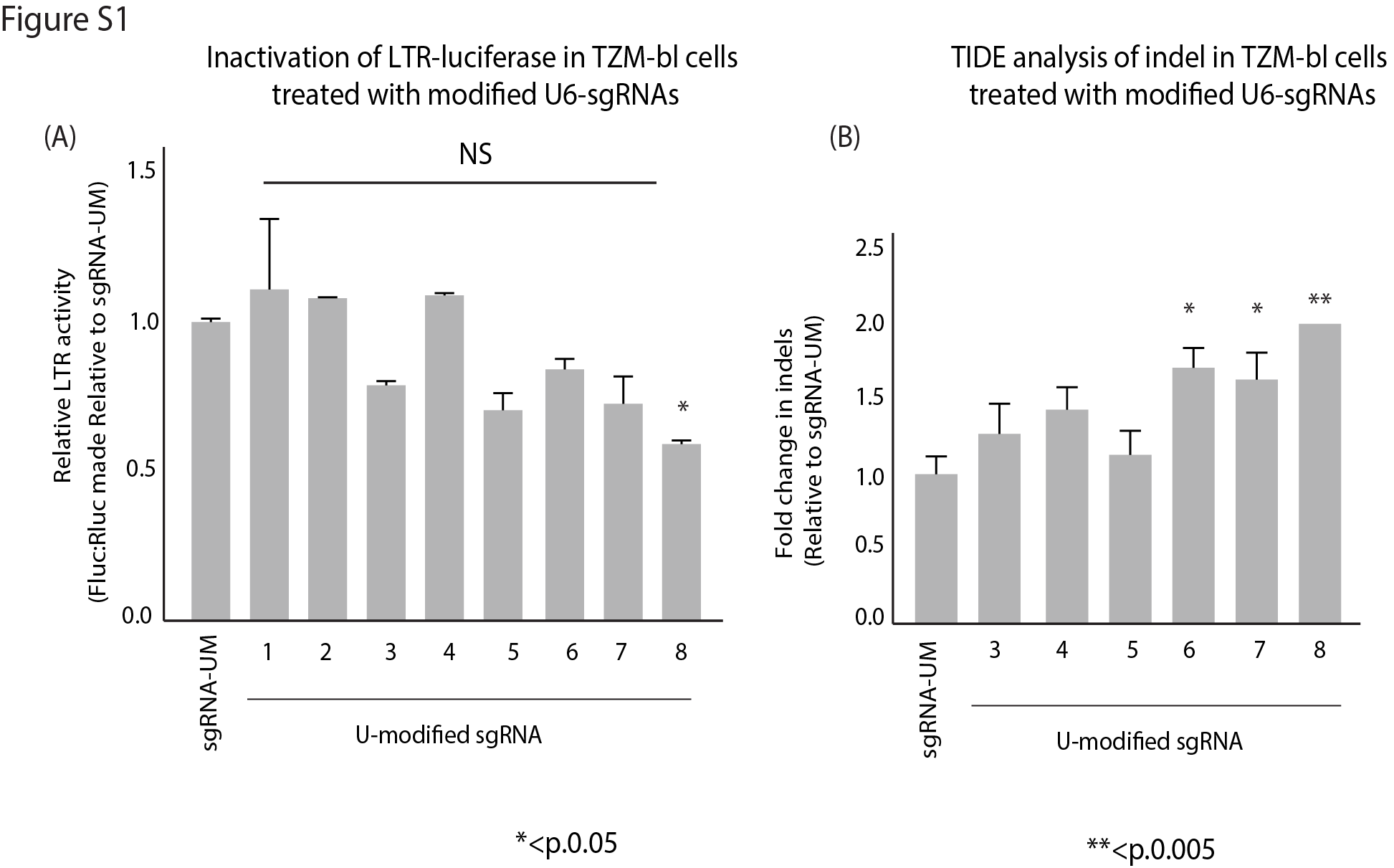


**Supp. Figure 1: Screening for U6 pol III sgRNAs that improved plasmid expressed CRISPR/Cas9 activity. (A)** A series of U-modified sgRNAs (1-8) expressed off U6 Pol III promoters were transfected with a Cas9 expression vector and *Renilla* background vector into TZM-bl cells. At 48 hrs post-transfection, the levels of luciferase was assessed by a Dual-luciferase assay. The levels of Firefly luciferase were normalized to *Renilla* luciferase and made relative to the unmodified control. (**B**) Total genomic DNA was extracted and the target sites was PCR amplified, and amplicons subjected to Sanger sequencing. The raw chromatograms were analyzed using the TIDE webtool. The errors bars represents standard error of the mean (SEM) of samples treated in duplicate. *p<0.05, **p<0.005 were obtained by one-way ANOVA and Dunnett's test.


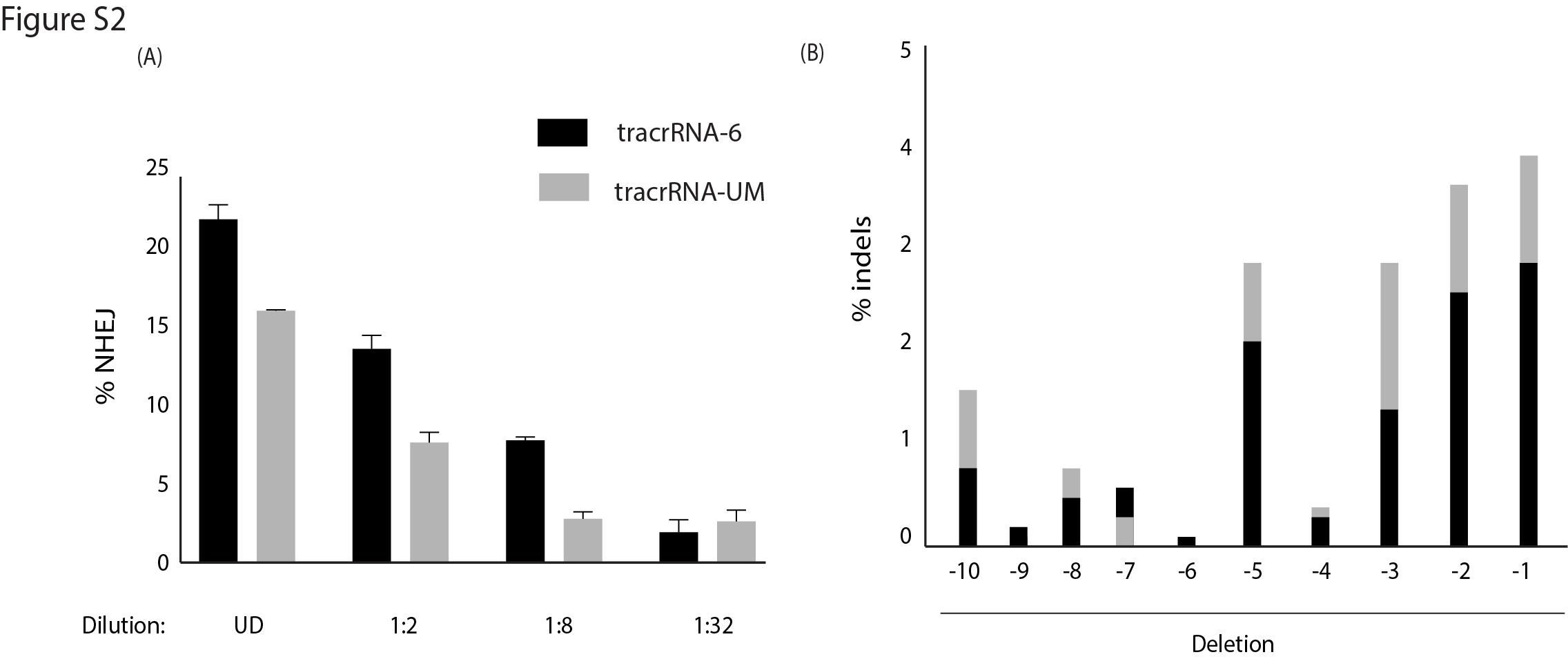


**Supp. Figure 2: TIDE analysis of U-modified tracrRNA.** Total genomic DNA was extracted from pMoHIV-C6 cells transfected with Cas9 sgRNAs containing tracrRNA-6 or tracrRNA-UM. The target site was PCR amplified and amplicons subjected to Sanger sequencing. The raw chromatograms were analysed using the TIDE webtool. **(A)** The total indel % and types of mutations **(B)** was determined. The errors bars represents standard error of the mean (SEM) of samples treated in duplicate, and the experiment was repeated twice.


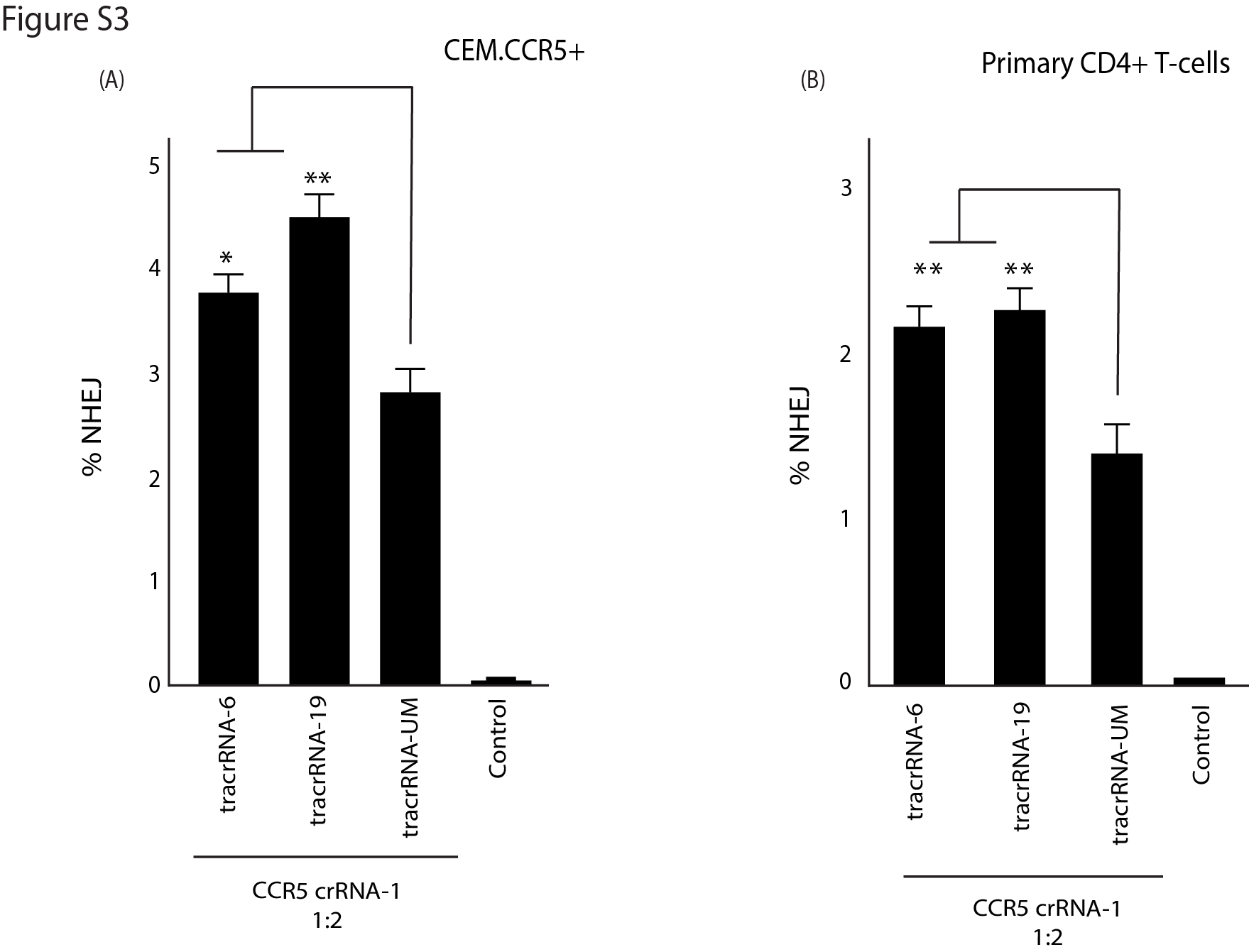


**Supp. Figure 3: The modified tracrRNAs improves indels at lower dilutions.** The tracrRNA-UM, tracrRNA-6 and tracrRNA-19 were annealed with a CCR5-crRNA 1, diluted 1_2 and electroporated into **(A)** CEM.CCR5+ cells and **(B)** primary CD4+ T-cells. The level of indel formation was measured using by a drop-off assay . The errors bars represent standard error of the mean (SEM)of samples treated in triplicate. *p<0.05,**p<0.005, were obtained by one-way ANOVA and Dunnett's test..


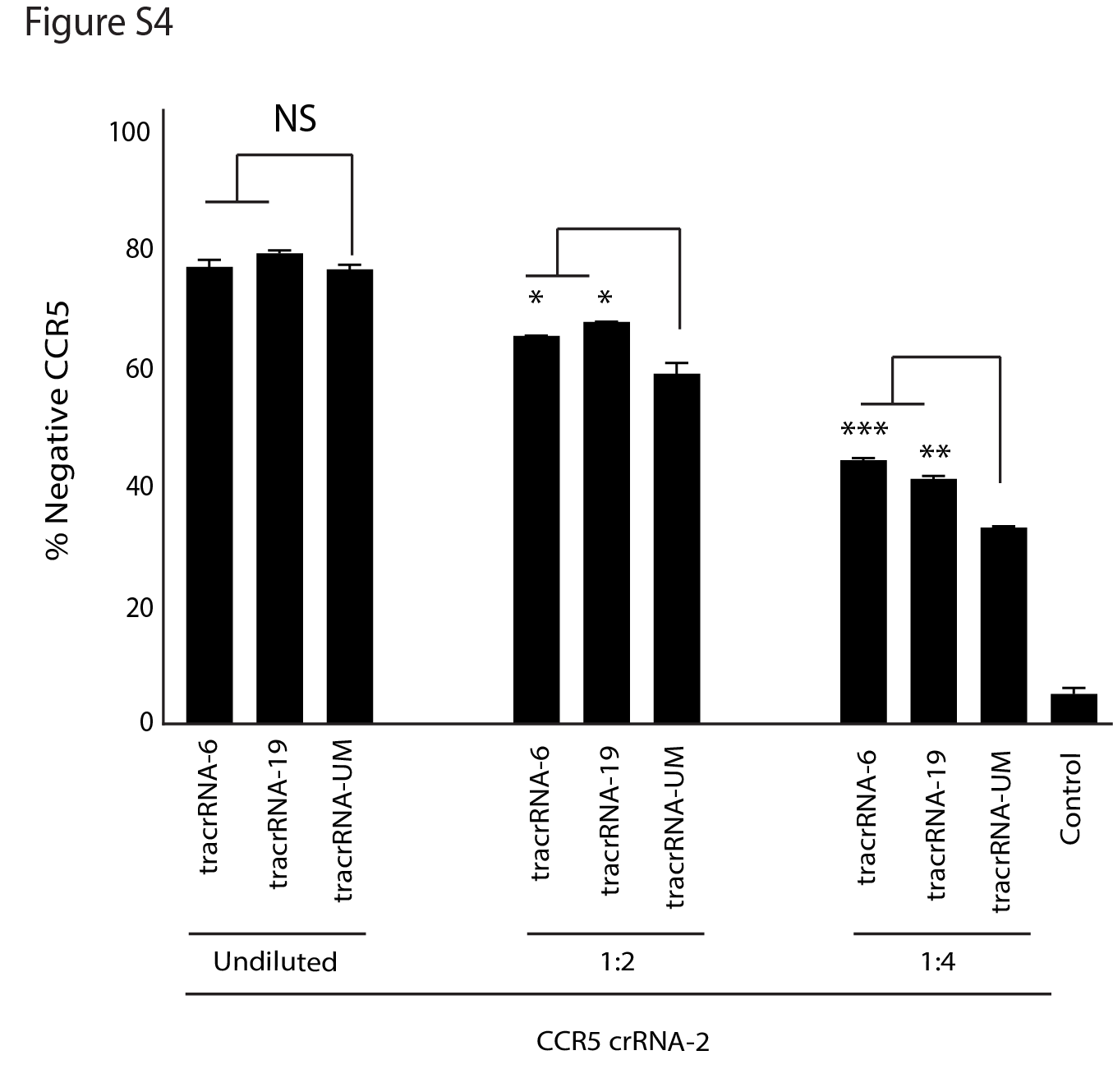


**Supp. Figure 4: The modified tracrRNAs improve CCR5 knockdown at lower dilutions.** The tracrRNA-UM, tracrRNA-6 and tracrRNA-19 were annealed with a CCR5-crRNA 2 and electroporated into CEM.CCR5+ cells either undiluted or at 1:2 or 1:4 dilutions. The level of CCR5 expression was assessed by FACS. The errors bars represent standard error of the mean (SEM) of samples treated in triplicate. *p<0.05, **p<0.005, ***p<0.001 were obtained by one-way ANOVA and Dunnett's test..


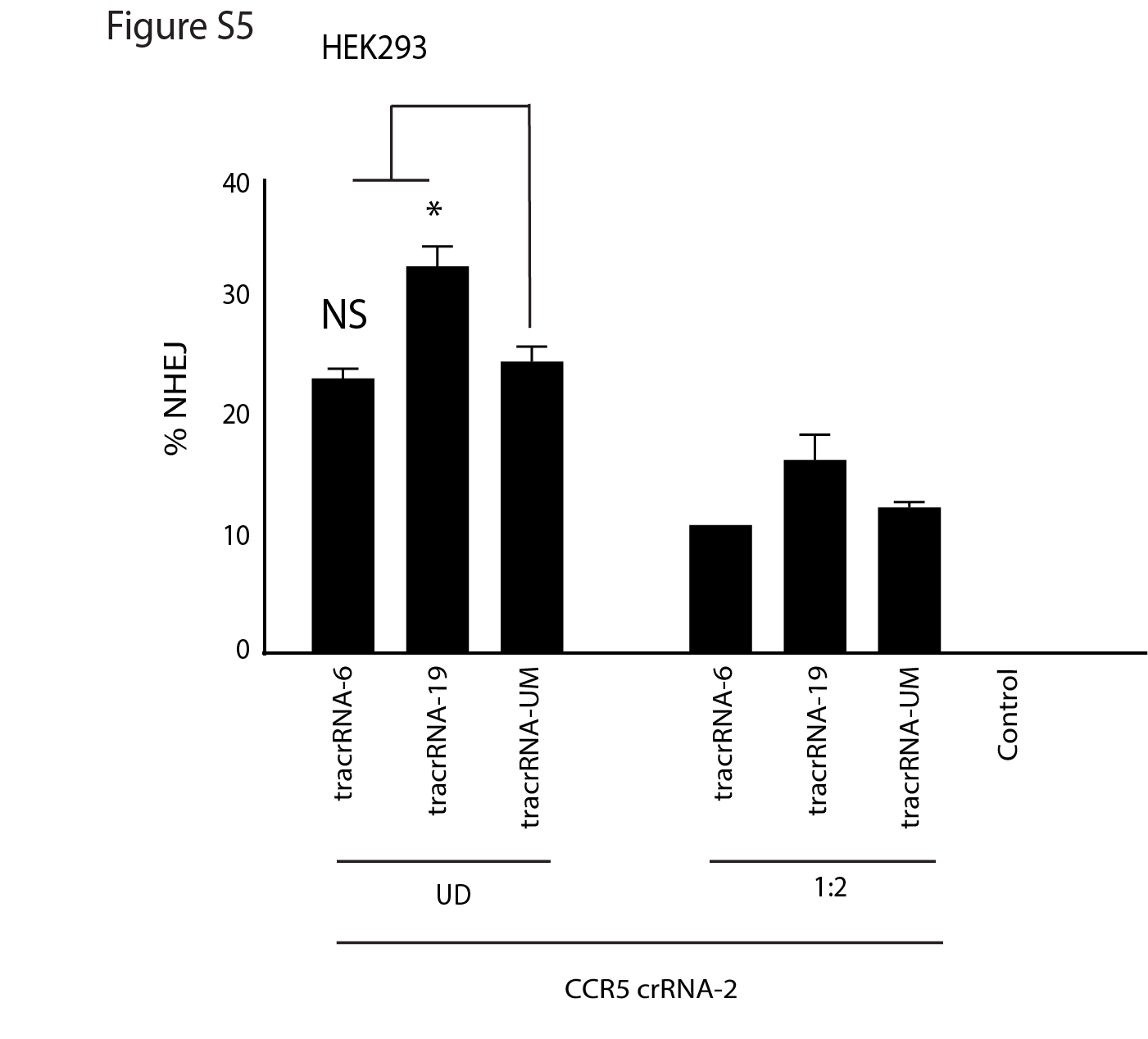


**Supp. Figure 5: The modified tracrRNAs improve indels at CCR5 target site in HEK293.** The tracrRNA-UM, tracrRNA-6 and tracrRNA-19 were annealed with a CCR5-crRNA 2 and electroporated into HEK293 cells undiluted or at a 1:2 dilution. The level of indels were measured by a drop-off assay. The errors bars represent standard error of the mean (SEM) of samples treated in triplicate. *p<0.05, were obtained by one-way ANOVA and Dunnett's test..

**Supplementary Table 1: Sequence of the U-modified U6-expressed sgRNAs.**

| sgRNA | **Sequence (5'-3')** |
| --- | --- |
| sgRNA-UM | **GAGAGCUCCCAGGCUCAGAUC**GUUUAAGAGCUAUGCUGGAAACAGCAUAGCAAGUUUAAAUAAGGCUAGUCCGUUAUCAACUUGAAAAAGUGGCACCGAGUCGGUGCUUU |
| U-modified sgRNA-1 | **GAGAGCUCCCAGGCUCAGAUC**GUUUAAGAGCUAUGCUGGAAACAGCAUAGCAAGaUUAAAUAAGGCUAGUCCGUUAUCAACUUGAAAAAGUGGCACCGAGUCGGUGCUUU |
| U-modified sgRNA-2 | **GAGAGCUCCCAGGCUCAGAUC**GUUUAAGAGCUAUGCUGGAAACAGCAUAGCAAGUgUAAAUAAGGCUAGUCCGUUAUCAACUUGAAAAAGUGGCACCGAGUCGGUGCUUU |
| U-modified sgRNA-3 | **GAGAGCUCCCAGGCUCAGAUC**GUUUAUGAGCUAUGCUGGAAACAGCAUAGCAAGUaUAAAUAAGGCUAGUCCGUUAUCAACUUGAAAAAGUGGCACCGAGUCGGUGCUUU |
| U-modified sgRNA-4 | **GAGAGCUCCCAGGCUCAGAUC**GUUUAAGAGCUAUGCUGGAAACAGCAUAGCAAGUUUAAAUAAGGCUAGUCCGaaAUCAACUUGAAAAAGUGGCACCGAGUCGGUGCUUU |
| U-modified sgRNA-5 | **GAGAGCUCCCAGGCUCAGAUC**GUUUAAGAGCUAUGCUGGAAACAGCAUAGCAAGUUUAAAUAAGGCUAGUCCGggAUCAACUUGAAAAAGUGGCACCGAGUCGGUGCUUU |
| U-modified sgRNA-6 | **GAGAGCUCCCAGGCUCAGAUC**GUUUAAGAGCUAUGCUGGAAACAGCAUAGCAAGUUUAAAUAAGGC_AGUCCGUUAUCAACUUGAAAAAGUGGCACCGAGUCGGUGCUUU |
| U-modified sgRNA-7 | **GAGAGCUCCCAGGCUCAGAUC**GUUUAAGAGCUAUGCUGGAAACAGCAUAGCAAGUUUAAAUAAGGCaAGUCCGUUAUCAACUUGAAAAAGUGGCACCGAGUCGGUGCUUU |
| U-modified sgRNA-8 | **GAGAGCUCCCAGGCUCAGAUC**GUUUAAGAGCUAUGCUGGAAACAGCAUAGCAAGUUUAAAUAAGGCUAGaCCGUUAUCAACUUGAAAAAGUGGCACCGAGUCGGUGCUUU |

Lower cases letters represent the U-modified sequence. The target sequence is highlighted in bold.

**Supp. Table 2: Sequence of crRNAs and tracrRNAs for U-modified tracrRNA screening**

| Modified RNA | **crRNA sequence (5'-3')** | **tracrRNA Sequence (5'-3')** |
| --- | --- | --- |
| tracrRNA-UM | **GAGAGCUCCCAGGCUCAGAUC**GUUUAAGAGCUAUGCU | AGCAUAGCAAGUUUAAAUAAGGCUAGUCCGUUAUCAACUUGAAAAAGUGGCACCGAGUCGGUGCUUU |
| tracrRNA-1 | **GAGAGCUCCCAGGCUCAGAUC**GUUUAuGAGCUAUGCU | AGCAUAGCAAGUaUAAAUAAGGCUAGUCCGUUAUCAACUUGAAAAAGUGGCACCGAGUCGGUGCUUU |
| tracrRNA -2 | **GAGAGCUCCCAGGCUCAGAUC**GUUUAuGAGCUuUGCU | AGCAaAGCAAGUaUAAAUAAGGCaAGaCCGggAUCAACUUGAAAAAGUGGCACCGAGUCGGUGCUUU |
| tracrRNA -3 | **GAGAGCUCCCAGGCUCAGAUC**GUUUAuGAGCUuUGCU | AGCAaAGCAAGUaUAAAUAAGGCaAGaCCGggAaCAACUUGAAAAAGUGGCACCGAGUCGGUGCUUU |
| tracrRNA -4 | **GAGAGCUCCCAGGCUCAGAUC**GUUUAAGAGCUuUGCU | AGCAaAGCAAGUUUAAAUAAGGCUAGUCCGUUAUCAACUUGAAAAAGUGGCACCGAGUCGGUGCUUU |
| tracrRNA -5 | **GAGAGCUCCCAGGCUCAGAUC**GUUUAuGAGCUAUGCU | AGCAUAGCAAGUaUAAAUAAGGCaAGaCCGggAaCAACUUGAAAAAGUGGCACCGAGUCGGUGCUUU |
| tracrRNA -6 | **GAGAGCUCCCAGGCUCAGAUC**GUUUAAGAGCUAUGCU | AGCAUAGCAAGUUUAAAUAAGGCUAGUCCGUUAaCAACUUGAAAAAGUGGCACCGAGUCGGUGCUUU |
| tracrRNA -7 | **GAGAGCUCCCAGGCUCAGAUC**GUUUAuGAGCUAUGCU | AGCAUAGCAAGUaUAAAUAAGGCaAGaCCGggAUCAACUUGAAAAAGUGGCACCGAGUCGGUGCUUU |
| tracrRNA -8 | **GAGAGCUCCCAGGCUCAGAUC**GUUUAAGAGCUAUGCU | AGCAUAGCAAGUUUAAAUAAGGCaAGUCCGUUAUCAACUUGAAAAAGUGGCACCGAGUCGGUGCUUU |
| tracrRNA -9 | **GAGAGCUCCCAGGCUCAGAUC**GUUUAAGAGCUAUGCU | AGCAUAGCAAGUUUAAAUAAGGCUAGUCCGggAUCAACUUGAAAAAGUGGCACCGAGUCGGUGCUUU |
| tracrRNA -10 | **GAGAGCUCCCAGGCUCAGAUC**GUUUAuGAGCUAUGCU | AGCAUAGCAAGUaUAAAUAAGGCaAGaCCGggAUCAACUUGAAAAAGUGGCACCGAGaCGGUGCUUU |
| tracrRNA -11 | **GAGAGCUCCCAGGCUCAGAUC**GUUUAuGAGCUAUGCU | AGCAUAGCAAGUaUAAAUAAGGCaAGaCCGggAUCAACUUGAAAAAGUGGCACCGAGgCGGUGCUUU |
| tracrRNA -12 | **GAGAGCUCCCAGGCUCAGAUC**GUUUAuGAGCUAUGCU | AGCAUAGCAAGUaUAAAUAAGGCUAGUCCGggAUCAACUUGAAAAAGUGGCACCGAGUCGGUGCUUU |
| tracrRNA -13 | **GAGAGCUCCCAGGCUCAGAUC**GUUUAuGAGCUAUGCU | AGCAUAGCAAGUaUAAAUAAGGCUAGaCCGggAUCAACUUGAAAAAGUGGCACCGAGUCGGUGCUUU |
| tracrRNA -14 | **GAGAGCUCCCAGGCUCAGAUC**GUUUAAGAGCUAUGCU | AGCAUAGCAAGUUUAAAUAAGGCUAGaCCGUUAUCAACUUGAAAAAGUGGCACCGAGUCGGUGCUUU |
| tracrRNA -15 | **GAGAGCUCCCAGGCUCAGAUC**GUUUAAGAGCUAUGCU | AGCAUAGCAAGUUUAAAUAAGGCUAGUCCGgUAUCAACUUGAAAAAGUGGCACCGAGUCGGUGCUUU |
| tracrRNA -16 | **GAGAGCUCCCAGGCUCAGAUC**GUUUAAGAGCUAUGCU | AGCAUAGCAAGUUUAAAUAAGGCUAGUCCGUgAUCAACUUGAAAAAGUGGCACCGAGUCGGUGCUUU |
| tracrRNA -17 | **GAGAGCUCCCAGGCUCAGAUC**GUUUAAGAGCUAUGCU | AGCAUAGCAAGUUUAAAUAAGGC_AGUCCGUUAUCAACUUGAAAAAGUGGCACCGAGUCGGUGCUUU |
| tracrRNA -18 | **GAGAGCUCCCAGGCUCAGAUC**GUUUAuGAGCUAUGCU | AGCAUAGCAAGUaUAAAUAAGGCUAGUCCGUUAaCAACUUGAAAAAGUGGCACCGAGUCGGUGCUUU |
| tracrRNA -19 | **GAGAGCUCCCAGGCUCAGAUC**GUUUAAGAGCUAUGCU | AGCAUAGCAAGUUUAAAUAAGGCUAGUCCGUUAaCAACggGAAAccGUGGCACCGAGUCGGUGCUUU |
| tracrRNA -20 | **GAGAGCUCCCAGGCUCAGAUC**GUUUAuGAGCUAUGCU | AGCAUAGCAAGUaUAAAUAAGGCUAGUCCGUgAaCAcCggGAAAccGgGGCACCGAGUCGGUGCUUU |
| tracrRNA -21 | **GAGAGCUCCCAGGCUCAGAUC**GUUUAAGAGCUAUGCU | AGCAUAGCAAGUUUAAAUAAGGCUAGUCCGUgAaCAcCggGAAAccGgGGCACCGAGUCGGUGCUUU |
| tracrRNA -22 | **GAGAGCUCCCAGGCUCAGAUC**GUUUAAGAGCUAUGCU | AGCAUAGCAAGUUUAAAUAAGGCUAGUCCGUUAaCAcCggGAAAccGgGGCACCGAGUCGGUGCUUU |
| tracrRNA -23 | **GAGAGCUCCCAGGCUCAGAUC**GUUUAAGAGCUAUGCU | AGCAUAGCAAGUUUAAAUAAGGCUAGUCCGUgAaCAACUUGAAAAAGUGGCACCGAGUCGGUGCUUU |

Lower cases letters represent the U-modified sequence. The Tar6 target sequence is highlighted in bold.

**Supplementary Table 3: TAR and CCR5 crRNA sequences.**

| **crRNA** | **sequence (5'-3')** |
| --- | --- |
| Tar3 crRNA | **GGUUAGACCAGAUCUGAGCC**GUUUAAGAGCUAUGCU |
| Tar4 crRNA | **GGGAGCUCUCUGGCUAACU**GUUUAAGAGCUAUGCU |
| Tar5 crRNA | **GUAACCAGAGAGACCCAGUAC**GUUUAAGAGCUAUGCU |
| CCR5 crRNA-1 | **ATAATTGCAGTAGCTCTAAC**GUUUAAGAGCUAUGCU |
| CCR5 crRNA-2 | **TAGAGCTACTGCAATTATTC**GUUUAAGAGCUAUGCU |
| HBB crRNA | \| **CUUGUCAAGGCUAUUGGUCA**GUUUAAGAGCUAUGCU \| \| --- \| \|  \| |
| BCL11A GATA crRNA | **CUAACAGUUGCUUUUAUCAC**GUUUAAGAGCUAUGCU |

The target sequence is highlighted in bold.

**Supplementary Table 4: Drop-off assay primer and probe sequences.**

| **oligomer** | **Sequence (5'-3')** | **nt** |
| --- | --- | --- |
| ddPCR TAR F | CGAGCCCTCAGATGCTACATA | 21 |
| ddPCR TAR R | TTTGAGCACTCAAGGCAAGC | 20 |
| TAR3/6 Target probe | FAM-TGGTTAGACCAGATCTGAGCCTGGGAGC-BHQ1 | 28 |
| TAR3/5/6 Reference probe | HEX-AGGCTTAAGCAGTGGGTTCCCTAGTTAGC-BHQ1 | 29 |
| TAR5 Target probe | FAM-TTGCCTGTACTGGGTCTCTCT-BHQ1 | 29 |
| TAR4 Target probe | FAM-TGGGTTCCCTAGTTAGCCAGAGA-BHQ1 | 23 |
| TAR4 Reference probe | HEX-TTGCCTGTACTGGGTCTCTCTGGT-BHQ1 | 24 |
| ddPCR CCR5 F | GGCTGTGAGGCTTATCTTCAC | 21 |
| ddPCR CCR5 R | TCTGTCACCTGCATAGCTTG | 21 |
| CCR5-1 Target probe | FAM-CAGTAGCTCTAACAGGTTGGACC-BHQ1 | 20 |
| CCR5-2 Target probe | FAM-CTACTGCAATTATTCAGGCCAAAG-BHQ1 | 24 |
| CCR5 Reference probe | HEX-TGGGCTCCCTACAACATTGTCCT-BHQ1 | 23 |
| HBB F | CTGGAGCTACAGACAAGAAGGTG | 23 |
| HBB R | CCACAGGCTTGTGATAGTAGCC | 22 |
| BCL11A GATA F | ACTGATGGACCTTGGGTGCTATT | 23 |
| BCL11A GATA R | GGGAAGCTTCACCTCCTTTACA | 22 |
